# The Interplay Between Thyroid Disorders and Diabetes and Their Impact on Cardiovascular Outcomes: A Systematic Review

**DOI:** 10.7759/cureus.93945

**Published:** 2025-10-06

**Authors:** Ajeeth Rehman Abdul Jaffar Azad, Zareen Zohara

**Affiliations:** 1 General Medicine, York and Scarborough Teaching Hospitals NHS Foundation Trust, York, GBR; 2 Trauma and Orthopaedics, West Suffolk Hospital, Bury St Edmunds, GBR

**Keywords:** cardiovascular illness, cardiovascular outcomes, diabetes, diabetes mellitus, hyperthyroidism, hypothyroidism, thyroid dysfunction

## Abstract

Diabetes mellitus and thyroid diseases are closely related, as both conditions share pathophysiological pathways and risk factors that increase cardiovascular risk. The purpose of this research is to thoroughly examine the literature about the connection between thyroid conditions and diabetes mellitus and determine how these conditions affect cardiovascular outcomes. PubMed, Google Scholar, Science Direct, and BioMed Central (BMC) databases were analysed using keywords "thyroid dysfunction", "hypothyroidism", "hyperthyroidism", "diabetes", "diabetes mellitus", "cardiovascular illness", and "cardiovascular outcomes". A comprehensive search of databases yielded studies published between 2011 and 2024, focusing on human subjects and the interplay between thyroid function and diabetes. The articles selected are screened for selection. A total of 4205 papers were selected after screening. After duplicate removal, 1185 articles were selected and underwent review, and 12 articles were chosen for quality appraisal using the Newcastle-Ottawa Scale for cross-sectional studies. Eight articles qualified the criteria for selection and quality appraisal. There is a bidirectional relationship between thyroid dysfunction and diabetes, underscoring the need for comprehensive management to mitigate cardiovascular risk found in this review. Regular thyroid screening, monitoring of cardiovascular risk factors, and timely intervention are crucial. Future research should prioritise longitudinal studies, standardised protocols, and molecular insights to inform evidence-based practice and optimise patient outcomes.

## Introduction and background

**Figure 1:**
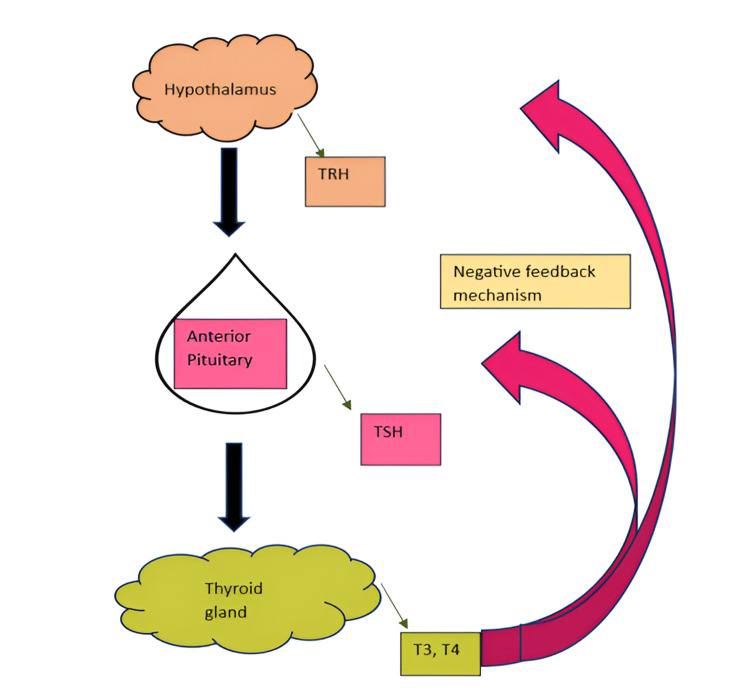
The hypothalamic pituitary axis and the negative feedback process TRH: thyrotropin-releasing hormone; TSH: thyroid-stimulating hormone Image credit: Created in Microsoft Word (Microsoft® Corp., Redmond, WA) by the co-author.

Thyroid hormone (TH) governs metabolic processes necessary for optimal growth and development, as well as metabolism [1]. Thyroid-stimulating hormone (TSH) is generated from the hypothalamus-pituitary-thyroid axis and is known to alter in accordance with the circadian rhythm [2]. Thyroxine (T4) and triiodothyronine (T3), two THs that are essential for controlling growth and metabolism, are synthesised and released when the hypothalamus produces thyroid-releasing hormone (TRH), which in turn triggers the anterior pituitary gland to produce TSH. Increased T3 and T4 levels limit TRH release in the hypothalamus and decrease TSH synthesis in the anterior pituitary [3], and vice versa, according to a negative feedback system that controls this. Figure 1 depicts the hypothalamic pituitary axis and the negative feedback process.

Any disruption in this mechanism leads to thyroid dysfunctions. The functions of the thyroid gland are of a wide range, which are simplified in Table 1.

**Table 1:** The functions of the thyroid gland ATP : Adenosine triphosphate

Study	Functions	
Shahid et al. [4]	Metabolism regulation	Regulate metabolic rate, influencing energy production and consumption.
Zoeller and Rovet [5]	Growth and development	Growth and development, especially in children and teenagers, depend on thyroid hormones.
Silva [6]	Energy production	Thyroid hormones stimulate the production of adenosine triphosphate (ATP), the powerhouse of the body.
de Souza [7]	Nervous system function	Thyroid hormones regulate neurotransmitter synthesis and release, influencing mood, cognitive function, and nervous system development
Yamakawa et al. [8]	Heart rate regulation	Thyroid hormones have numerous effects on the cardiovascular and peripheral vascular systems. It is well acknowledged that they increase heart rate and cardiac contractility, improve systolic and diastolic function, and reduce systemic vascular resistance (SVR) during rest.
Bassett and Williams [9]	Bone health	Thyroid hormones control osteoclast activity, which influences bone resorption and density.
Silva et al., Krassas et al. [10,11]	Reproductive function	Thyroid hormones regulate menstrual cycle regularity, fertility, and pregnancy maintenance.
Safer [12]	Skin, hair, and nail health	Thyroid hormones influence skin, hair, and nail growth and maintenance.

Any change in the TH production results in a disruption in the functions they perform. TSH levels in adults are considered within the normal range when they fall between 0.4 and 4.0 mIU/mL [13]. Depending on the range, thyroid dysfunction is subdivided into hypothyroid, hyperthyroid, and subclinical thyroid. The thyroid function test is used to interpret and differentiate between them.

**Figure 2:**
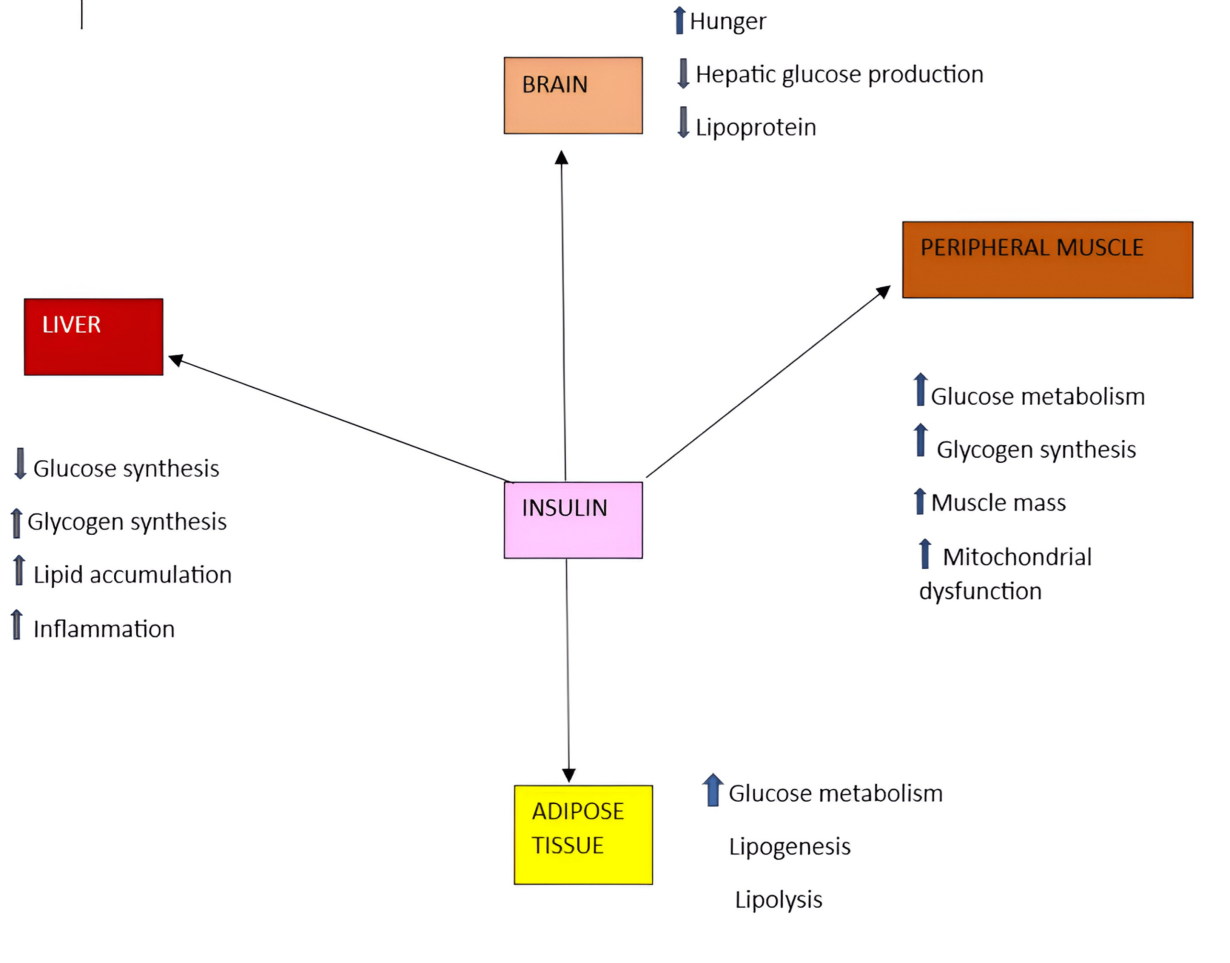
The physiological function of insulin Image credit: Created in Microsoft Word (Microsoft® Corp., Redmond, WA) by the co-author.

Diabetes affects 537 million people aged 20 to 79, accounting for one in 10. The figure is predicted to rise to 643 million by 2030, and 783 million by 2045 [14]. Diabetes mellitus (DM) is defined principally by elevated blood glucose levels (hyperglycaemia), polydipsia, and polyphagia [15]. Diabetes can be classified into several subtypes: type 1, type 2, MODY, gestational, neonatal, and steroid-induced diabetes [16]. The most common type of diabetes is type 2 diabetes mellitus (T2DM), which is characterised by insufficient insulin secretion or insulin receptor desensitisation, which prevents glucose from entering the cells [17]. Glucose homeostasis is largely governed by insulin's actions. The insulin is produced from the β-cells of the islets of Langerhans [16]. The physiological function of insulin is depicted below in Figure 2 [18].

The mechanism behind T2DM is explained in detail, considering it is the most prevalent. Skeletal muscle, liver, and adipose tissue are the three main sites resistant to insulin. When the circulating glucose from carbohydrate is in excess, it results in the resistance of the tissues in the body. Approximately 70% of glucose excretion takes place in skeletal muscle, which makes it a significant reservoir of circulating glucose. Reduced muscle tissue absorption of glucose is a result of muscle insulin resistance. Glucose is transported from the muscle to the liver, where de novo lipogenesis (DNL) takes place. With an increase in glucose substrate, the liver develops insulin resistance. Higher rates of DNL raise plasma triglyceride levels and generate an environment of surplus energy substrate. This promotes insulin resistance throughout the body, contributing to ectopic lipid deposition in and surrounding visceral organs [19].

The American Diabetes Association prefers fasting plasma glucose (FPG) values to the oral glucose tolerance test (OGTT) for diagnosing DM. The diagnostic cut-off for FPG is 126 mg/dL (7.0 mmol/L). Indicators of diabetes include a random plasma glucose level of 200 mg/dL (11.1 mmol/L), a two-hour plasma glucose level of 200 mg/dL (11.1 mmol/L) or higher during an OGTT, and a HbA1c level of 6.5% or higher [20]. The haemoglobin A1c (glycated haemoglobin, glycosylated haemoglobin, HbA1c, or A1c) test assesses a person's level of glucose management. The test calculates the average blood sugar level over the last 90 days and reflects it as a percentage. This test can also be used to diagnose diabetes [21].

Individuals with T2DM are more likely to develop TD than the general population [22]. The prevalence of TD in T2DM varies per study, ranging from extremely low (5.5%) to very high (75%) [23]. The nocturnal TSH peak is blunted or eliminated in DM patients, and the hypothalamic TSH response to TRH is compromised, resulting in hypothyroidism. These mechanisms are thought to be responsible for the increased risk of thyroid disease in DM patients, particularly those with poor glycaemic control [24]. The pathophysiological link between T2DM and TD is assumed to be the result of an interplay between multiple biochemical, genetic, and hormonal abnormalities [25].

TH excess and deficiency often increase the risk of early morbidity and death by causing or exacerbating cardiovascular disease (CVD) such as atrial and ventricular arrhythmias, atherosclerotic vascular disease, dyslipidaemia, and heart failure [26]. Though there are various studies analysing the prevalence of thyroid disorder (TD) in DM, few studies have thoroughly investigated the triadic relationship between thyroid dysfunction (TD), T2DM, and CVD, despite the fact that numerous studies have investigated the prevalence of TD in DM. By combining data on the effects of TD on cardiovascular outcomes and glycaemic management in people with T2DM, this systematic review seeks to close that gap. Our review differs from earlier research that usually looks into these disorders separately because of this distinction.

## Review

Methods

In this systematic review, we have followed the Preferred Reporting Items for Systematic Reviews and Meta-Analyses (PRISMA) 2020 standards [27]. PubMed, Google Scholar, Science Direct, BioMed Central (BMC), and Cochrane are the search engines for the database. The last search was done on October 23, 2024. Keywords were thyroid dysfunction, hypothyroidism, hyperthyroidism, diabetes, DM, cardiovascular illness, and cardiovascular outcomes. The keywords used were found using a Medical Subject Headings (MeSH) search. We found 4205 eligible articles across all the databases. Table 2 summarises the search strategies utilised in various journals.

**Table 2:** A summary of search strategies used in PubMed, Google Scholar, Science Direct, BioMed Central (BMC), and Cochrane

Database	Keywords	Search Strategy	Filters Used	Results
PubMed	"Thyroid dysfunction”, "hypothyroidism”, “hyperthyroidism”, “diabetes”, “diabetes mellitus”, “cardiovascular disease”, “cardiovascular outcomes"	("Diabetes mellitus/blood" [MeSH] OR "diabetes mellitus/chemically induced" [MeSH] OR "diabetes mellitus/classification" [MeSH] OR "diabetes mellitus/congenital" [MeSH] OR "diabetes mellitus/diagnosis" [MeSH] OR "diabetes mellitus/diet therapy" [MeSH] OR "diabetes mellitus/drug therapy" [MeSH] OR "diabetes mellitus/epidemiology" [MeSH] OR "diabetes mellitus/etiology" [MeSH] OR "diabetes mellitus/genetics" [MeSH] OR "diabetes mellitus/history" [MeSH] OR "diabetes mellitus/immunology" [MeSH] OR "diabetes mellitus/metabolism" [MeSH] OR "diabetes mellitus/pathology" [MeSH] OR "diabetes mellitus/physiopathology" [MeSH] OR "diabetes mellitus/prevention and control" [MeSH] OR "diabetes mellitus/rehabilitation" [MeSH] OR "diabetes mellitus/therapy" [MeSH] ) AND ("thyroid diseases/blood" [MeSH] OR "thyroid diseases/classification" [MeSH] OR "thyroid diseases/complications" [MeSH] OR "thyroid diseases/diagnosis" [MeSH] OR "thyroid diseases/diagnostic imaging" [MeSH] OR "thyroid diseases/diet therapy" [MeSH] OR "thyroid diseases/epidemiology" [MeSH] OR "thyroid diseases/etiology" [MeSH] OR "thyroid diseases/genetics" [MeSH] OR "thyroid diseases/history" [MeSH] OR "thyroid diseases/metabolism" [MeSH] OR "thyroid diseases/pathology" [MeSH] OR "thyroid diseases/physiopathology" [MeSH] AND ("cardiovascular diseases/blood" [MeSH] OR "cardiovascular diseases/classification" [MeSH] OR "cardiovascular diseases/complications" [MeSH] OR "cardiovascular diseases/diagnosis" [MeSH] OR "cardiovascular diseases/diagnostic imaging" [MeSH] OR "cardiovascular diseases/drug therapy" [MeSH] OR "cardiovascular diseases/enzymology" [MeSH] OR "cardiovascular diseases/ethnology" [MeSH] OR "cardiovascular diseases/etiology" [MeSH] OR "cardiovascular diseases/history" [MeSH] OR "cardiovascular diseases/immunology" [MeSH] OR "cardiovascular diseases/metabolism" [MeSH] OR "cardiovascular diseases/mortality" [MeSH] OR "cardiovascular diseases/pathology" [MeSH] OR "cardiovascular diseases/physiopathology" [MeSH] OR "cardiovascular diseases/prevention and control" [MeSH])	2014-2024	1524
Google Scholar	"Thyroid dysfunction”, "hypothyroidism”, “hyperthyroidism”, “diabetes”, “diabetes mellitus”, “cardiovascular disease”, “cardiovascular outcomes"	Thyroid dysfunction, hyperthyroidism, diabetes mellitus, cardiovascular disease, cardiovascular outcomes	Simply using the title and all the keywords in an advanced search	2660
Science Direct	"Thyroid dysfunction”, "hypothyroidism”, “hyperthyroidism”, “diabetes”, “diabetes mellitus”, “cardiovascular disease”, “cardiovascular outcomes"	Thyroid dysfunction, hyperthyroidism, diabetes mellitus, cardiovascular disease, cardiovascular outcomes	No filters used	9
BMC	"Thyroid dysfunction”, "hypothyroidism”, “hyperthyroidism”, “diabetes”, “diabetes mellitus”, “cardiovascular disease”, “cardiovascular outcomes"	Thyroid dysfunction, hyperthyroidism, diabetes mellitus, cardiovascular disease, cardiovascular outcomes	No filters used	5
Cochrane	"Thyroid dysfunction”, "hypothyroidism”, “hyperthyroidism”, “diabetes”, “diabetes mellitus”, “cardiovascular disease”, “cardiovascular outcomes"	Thyroid dysfunction, hyperthyroidism, diabetes mellitus, cardiovascular disease, cardiovascular outcomes	No filters used	7

Eligibility Criteria

The studies that were selected are based on the participants and outcomes. With participants with thyroid dysfunction from all ethnicities, age >18, and genders with underlying diabetes. The outcomes are cardiovascular events (e.g., myocardial infarction, stroke), cardiovascular mortality, hospitalisation for heart failure, or atherosclerotic disease progression.

Inclusion Criteria

Studies comparing thyroid dysfunction with cardiovascular outcomes or the impact of TD treatment were included in this systematic review. The study population was not restricted based on gender or ethnicity. Only articles in English and full-text open-access journals, including human participants, and published from October 23, 2011, to October 23, 2024. Furthermore, the study incorporated observational studies, meta-analyses, literature, systematic reviews, and randomised controlled trials (RCTs).

Exclusion Criteria

Studies were excluded if they involved animals, non-diabetic studies, or focused on pregnant/lactating women, type 1 diabetes, or paediatric TDs. Case reports, unpublished works, editorials, and pre-2011 publications were also omitted. Our systematic review included studies meeting PICO criteria, specifically examining thyroid dysfunction's impact on cardiovascular outcomes in adults with diabetes, comparing thyroid-cardiovascular associations, and reporting relevant events, mortality, hospitalisation, or disease progression.

*Data Selection* 

The relevant studies were selected and retrieved independently by the authors. The two researchers discussed the study design, intervention implementation, outcomes measured, and relevance to our inclusion and exclusion criteria. Twelve articles were chosen from the five databases after duplicates were deleted using EndNote (Clarivate, Philadelphia, PA) software. We used the inclusion and exclusion criteria with the search strategy. Both authors discussed the reasons for differing opinions, referring back to the inclusion/exclusion criteria, and ensured a shared understanding of the study's relevance to the research question was made; all final decisions were made after clear discussion. The researchers evaluated the papers throughout the screening phase, and seven articles were chosen for quality assessment. These 12 articles were tested for quality appraisal.

Quality Assessment

The 12 articles selected were assessed for quality assessment using the Newcastle-Ottawa Scale for cross-sectional studies articles [28,29]. The selected studies were those which has more than 70% score and are shown in Table 3.

**Table 3:** The selected articles after quality appraisal

Studies approved after the review	Study type	Quality appraisal tool	Total score	Study score
Sarfo-Kantanka et al. [30]	Cross-sectional study	Newcastle-Ottawa Scale	9	7
Gweeq et al. [31]	Cross-sectional study	Newcastle-Ottawa Scale	9	7
Wang et al. [32]	Cross-sectional study	Newcastle-Ottawa Scale	9	7
Chen et al. [33]	Cross-sectional study	Newcastle-Ottawa Scale	9	7
Kesani et al. [34]	Cross-sectional study	Newcastle-Ottawa Scale	9	7
Arunkumar et al. [35]	Cross-sectional study	Newcastle-Ottawa Scale	9	7
Chubb et al. [36]	Cohort study	Newcastle-Ottawa Scale	9	7
Shah et al. [37]	Cohort study	Newcastle-Ottawa Scale	9	8

Results

**Figure 3:**
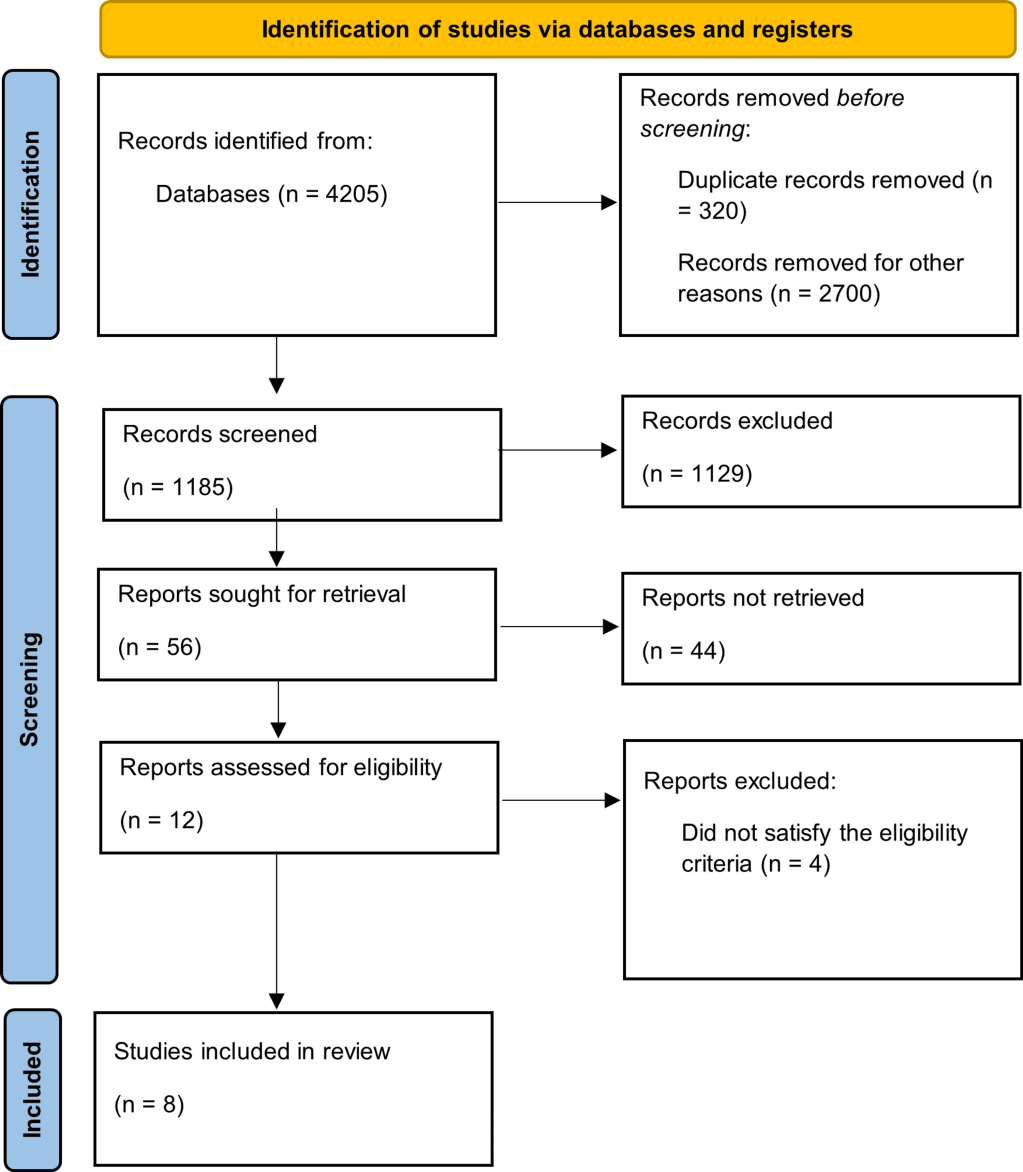
Flowchart of the Preferred Reporting Items for Systematic Reviews and Meta-Analyses (PRISMA)

Groups with TDs and cardiovascular outcomes with a history of diabetes were screened across all five databases. Initially, the search results showed 10777 papers. We narrowed down our search to 4205 publications after applying a number of filters, including the English language, free full text, keywords in the title, and our inclusion/exclusion criteria. Before the studies were selected and evaluated, 320 duplicates were removed both manually and using EndNote. A total of 2700 other articles were removed, which were not clear, articles outside the scope, and conference abstracts. The titles and abstracts of the 1185 publications were carefully examined to see if they were relevant to our review. A total of 1129 of the screened items were removed because they did not relate to the topic, objectives, inclusion, or exclusion criteria. Twelve papers were selected for quality evaluation as a result. Only eight articles were included in the final study that met the inclusion criteria due to one or more of the following: failure to meet minimum quality thresholds per quality appraisal, lack of direct relevance to the research question, inadequate methodological transparency, or insufficient data reporting for synthesis. This procedure is shown in the flowchart below (Figure 3).

A total of eight articles made it to the final review [30-37]. Table 4 contains the details of the relevant eight articles included in the review.

**Table 4:** Details of the relevant eight articles after quality appraisal BMI: body mass index; CAD: coronary artery disease; CHD: coronary heart disease; DMH: diabetes mellitus and hypothyroidism; ECG: electrocardiogram; HbA1c: glycated haemoglobin; LDL: low-density lipoprotein; T2DM: type 2 diabetes mellitus; TSH: thyroid-stimulating hormone

Serial number	Author	Year	Type	Purpose	Result	Conclusion
1	Sarfo-Kantanka et al. [30]	2018	Cross-sectional study	To determine how thyroid malfunction affects patients with T2DM cardiovascular risk	A study of 780 T2DM patients found a significant correlation between hypothyroidism and increased cardiovascular disease (CHD) risk. The median 10-year CHD score was higher in males (12) than females (9.4). Positive correlations were found between CHD risk and factors including HbA1c, total cholesterol, LDL cholesterol, systolic blood pressure, and thyroid-stimulating hormone (TSH). These findings emphasise the importance of managing traditional cardiovascular risk factors and suggest a potential role of thyroid function in CHD risk assessment.	The presence of thyroid dysfunction significantly increased the CHD risk associated with T2DM patients in Ghana.
2	Gweeq et al. [31]	2022	Cross-sectional study	The current study sought to identify the prevalence of thyroid dysfunction in T2DM patients attending the diabetes outpatient clinic at Mansoura Medical Specialized Hospital, as well as its relationship to cardiovascular risk factors.	A study of T2DM patients found those with hypothyroidism (group 1) had higher BMI, blood pressure, and cholesterol, and an average nine-year diabetes duration. In contrast, patients without hypothyroidism (group 2) had healthier thyroid function, with 90% having normal thyroid levels. Group 1 also had more subclinical hypothyroidism, indicating a link between hypothyroidism and worse health outcomes in T2DM patients.	It could be concluded that thyroid dysfunction is frequently developed among patients with T2DM. Screening for the thyroid functions among diabetic patients is needed to prevent cardiovascular risks.
3	Wang et al. [32]	2020	Cross-sectional study	To investigate the association of thyroid hormone levels with the risk of developing atherosclerosis in euthyroid T2D patients in Central China.	A total of 373 of the 910 participants had an atherosclerosis diagnosis. This study includes 387 male participants and 523 female participants; 51.9 was the average age. The BMI was 25.3 kg/m^2^ on average. A significant prevalence of atherosclerosis was linked to low-normal blood levels of free triiodothyronine (FT3) (3.50-4.17 pmol/L). In patients with mid- (4.17-4.83 pmol/L) and high-normal (4.83-6.50 pmol/L) FT3 levels, the prevalence ratio is 0.74 (95% CI 0.56 to 0.97, p = 0.029) and 0.63 (95% CI 0.46 to 0.87, p = 0.005), respectively, when compared to low-normal FT3. A higher free thyroxine (FT4) level was likewise associated with a lower incidence of atherosclerosis. The FT3 to FT4 ratio and thyroid-stimulating hormone (TSH) did not significantly correlate with the onset of atherosclerosis.	T2D patients with low but clinically normal FT3 level are more likely to develop macrovascular complications comparing with those with mid- and high-normal FT3 level.
4	Chen et al. [33]	2007	Cross-sectional study	The aim of this study is to investigate whether subclinical hypothyroidism is a risk factor for kidney disease and cardiovascular diseases.	Compared to euthyroid diabetics, subclinical hypothyroidism was linked to a higher incidence of diabetic nephropathy (odds ratio, 3.15 (95% CI, 1.48-6.69)). A total of 51 patients experienced cardiovascular events over the course of the follow-up period, which lasted 44.0 ± 7.4 months. After controlling for age, sex, A1C, other common cardiovascular risk factors, and medication, the risk of cardiovascular events was significantly elevated in Type 2 diabetics with subclinical hypothyroidism (hazard ratio, 2.93; 95% CI, 1.15-7.48; p = 0.024). There was no discernible difference between thyroid function and the rates of cardiovascular-related and overall death.	T2DM patients with subclinical hypothyroidism have an elevated risk of nephropathy and cardiovascular problems.
5	Kesani et al. [34]	2003	Cross-sectional study	"To determine the association between subclinical hypothyroidism and the risk of kidney disease and cardiovascular disease in T2DM patients."	Significant three-vessel CAD was found in nine out of 10 patients with diabetes plus hypothyroidism, 10 out of 25 patients with diabetes without hypothyroidism, and 10 out of 65 patients (15%) with no diabetes or hypothyroidism.	Three-vessel or four-vessel CAD was substantially more common in persons with diabetes mellitus and hypothyroidism than in those with diabetes mellitus and neither condition but without hypothyroidism.
6	Arunkumar et al. [35]	2017	Cross-sectional study	The study aimed to confirm the link between these two conditions and cardiovascular diseases and to quantify the incidence of hypothyroidism among individuals with T2DM.	The comparative analysis of 208 T2DM patients, divided into DM and DMH categories, uncovered notable differences in biochemical profiles. Specifically, the DM category exhibited elevated LDL levels, decreased HDL levels, and increased total cholesterol in the DC subgroup. Conversely, the DMH category displayed higher LDL levels and lower HDL levels. Furthermore, while the DM category showed no correlation with blood pressure or electrocardiogram parameters, the DMH category revealed significant associations. Additionally, intergroup comparisons yielded statistically significant differences in serum TSH, total cholesterol, and LDL levels, but not in FBS, PPBS, triglycerides, or HDL levels.	Patients with diabetes and hypothyroidism (DMH) had poor glycaemic control, higher lipid profiles, higher TSH levels and association with blood pressure and ECG abnormalities compared to patients with diabetes alone (DM).
7	Chubb et al. [36]	2022	Cohort	The aim of this study was to determine whether undetected thyroid disease increases the risk of incident CVD and death in T2DM.	The majority of individuals with newly diagnosed thyroid impairment (77.2%) had subclinical hypothyroidism, whereas overt/subclinical thyrotoxicosis was rare. Incident myocardial infarction were not more common in individuals with TSH > 5.1 mU/L than in those with TSH 0.34-2.9 mU/L. Subclinical hypothyroidism was independently correlated with serum systolic blood pressure and estimated glomerular filtration rate at baseline.	Subclinical hypothyroidism was not independently associated with CVD events or mortality in community-dwelling people with T2DM despite its associations with CVD risk factors, questioning strategies to identify and/or treat mild thyroid dysfunction outside usual care.
8	Shah et al. [37]	2023	Cohort	Our objective in this study was to assess the association of thyroid function and thyroid hormone replacement with cardiovascular outcomes in high-risk individuals with dysglycaemia and additional cardiovascular risk factors.	2.2% of patients had overt hypothyroidism, 5.5% had subclinical hypothyroidism, 0.8% had hyperthyroidism, and 91.5% were euthyroid. Hypothyroidism that was subclinical predicted both the enlarged cardiovascular outcome.	Subclinical hypothyroidism predicts future cardiovascular events and mortality in people with dysglycaemia and other cardiovascular risk factors.

Discussion

The complex link between the thyroid gland and DM has received a lot of study in recent years. Thyroid illnesses, particularly hypothyroidism (underactive thyroid) and hyperthyroidism (overactive thyroid), can have a major impact on glucose metabolism and insulin sensitivity, worsening or even causing diabetes. Diabetes, on the other hand, can disrupt thyroid function, resulting in a complex interaction between these two endocrine systems. This study investigates the relationship between thyroid diseases and diabetes, as well as the risk factors for both and their effects on cardiac outcomes, which includes the association between thyroid issue subtypes and cardiovascular risk factors such as dyslipidaemia, hyperglycaemia, and hypertension. Many recent studies have only found an association between thyroid problems and DM. The included studies contributed to a deeper understanding of the multifaceted interplay and its various consequences.

Relationship Between Thyroid Disorder and Diabetes Mellitus

Did you know that one in five diabetic patients has an undiagnosed TD? Research reveals a shocking 13.2% to 47.6% prevalence rate [22,38-41], with women being disproportionately affected [39-41]. But what is behind this alarming link? Shared risk factors like autoimmunity, inflammation, oxidative stress, and genetic predisposition create a perfect storm [41-45]. Anti-thyroid peroxidase antibodies are commonly identified in people with T2DM [46], while chronic inflammation and oxidative stress damage thyroid tissue, altering hormone synthesis [41,42,45]. Genetic variations linked to thyroid dysfunction and diabetes also play a role [47]. Smoking, advanced age, poor physical activity, high waist circumference, poor HDL cholesterol, and elevated triglycerides add to the risk [22,39,40].

Pathophysiology Behind the Interplay Between Diabetes and Thyroid Disorder

**Figure 4:**
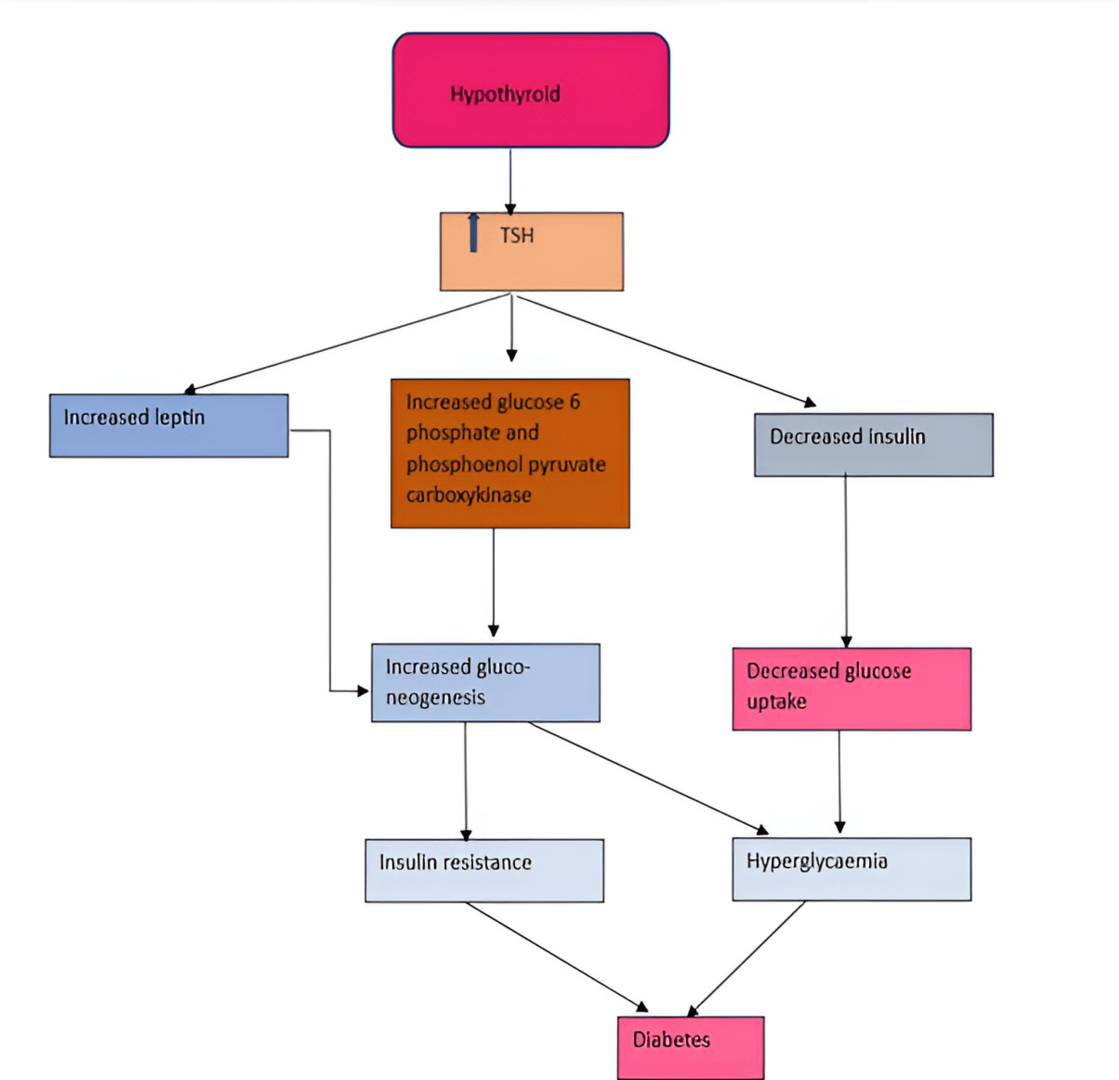
The mechanism by which hypothyroidism causes diabetes Image credit: Dr. Ajeeth Rehman, created in Microsoft Word (Microsoft® Corp., Redmond, WA).

The thyroid and pancreas are intimately linked. Emerging research reveals a bidirectional bond between thyroid dysfunction and DM, sparking a vicious cycle of metabolic mayhem. Hypothyroidism and hyperthyroidism disrupt glucose balance, fuelling insulin resistance and T2DM. Meanwhile, diabetes wreaks havoc on thyroid function, altering hormone levels, gland structure, and autoimmune responses. Understanding this intricate relationship can revolutionise the approach to managing blood sugar and thyroid health. The mechanism by which hypothyroidism causes diabetes is depicted in Figure 4 [48].

Likewise, hyperthyroidism also contributes to diabetes. Increased glucose production from the liver is the primary cause of hyperinsulinemia, glucose intolerance, and the development of peripheral insulin resistance. Glucose tolerance in thyrotoxicosis is characterised by increased hepatic glucose production and glycogenolysis. This process is responsible for the progression of subclinical diabetes and the amplification of hyperglycaemia in T2DM. Thyrotoxicosis may cause ketoacidosis due to enhanced lipolysis and hepatic β oxidation [49].

Studies have also found that T2DM and hyperthyroidism share several pathogenic characteristics. TD2M exhibits a unique pathophysiological signature, characterised by disrupted insulin secretion, altered glucose handling, and heightened catecholamine levels, which synergise to promote hyperthyroidism [50].

Impact of Type 2 Diabetes on Thyroid Disorders

T2DM increases the risk of developing hypothyroidism. Diabetes reduces thyroid function by altering TSH levels and disrupting the conversion of thyroxine (T4) to triiodothyronine (T3) in peripheral tissues [51]. When interpreting thyroid function tests in diabetic patients, consider the impact of diabetic ketoacidosis (DKA) and hyperinsulinemia/insulin resistance. DKA decreases T3 and T4 levels while maintaining normal or slightly elevated TSH levels. Goitre formation is promoted by insulin resistance, which aids thyroid tissue growth [52].

Cardiovascular Consequences of Thyroid Disorders in Diabetes

Breakthrough research reveals a shocking link between thyroid malfunction and diabetes, putting millions at risk for cardiovascular complications. Eight pivotal studies uncover the alarming truth: hypothyroidism significantly increases CVD risk in T2DM patients, with males facing a higher risk [30]. T2DM patients with hypothyroidism have higher BMI, blood pressure, and cholesterol levels, making thyroid screening crucial for preventing CVD [31]. Low FT3 levels are linked to atherosclerosis, while higher FT4 levels lower the risk [32]. Subclinical hypothyroidism raises the risk of cardiovascular problems [33] and worsens glycaemic control, lipid profiles, and blood pressure in diabetic patients [35]. Although subclinical hypothyroidism isn't independently linked to CVD events in T2DM patients [36], for people with dysglycaemia and other risk factors, it forecasts future cardiovascular events and death [37]. These findings underscore the importance of managing traditional CVD risk factors and highlight thyroid function's potential role in CHD risk assessment, sounding a critical alarm for early detection and intervention.

HbA1c: The Silent Narrator

HbA1c, or glycated haemoglobin, forms when glucose in the body adheres to red blood cells, accumulating in the blood due to impaired glucose processing. As red blood cells remain active for approximately two to three months, HbA1c readings are taken quarterly to reflect average blood glucose levels over this period [53]. Notably, non-diabetic individuals with subclinical hypothyroidism have been found to have elevated serum HbA1c levels [54-56]. 

The nature of this association - whether it happens independently of glycaemic control or is influenced by insulin resistance - is unknown. Bhattacharjee et al. conducted a cross-sectional study comparing HbA1c values between euthyroid, overt hypothyroid, and subclinical hypothyroid groups, and found that the SCH group had significantly higher HbA1c despite normal fasting glucose and no known diabetes [55].

However, the study did not include insulin sensitivity indicators like HOMA-IR, which limits the capacity to rule out insulin resistance as a confounder. Similarly, Ponnudhali and Preetha [54] found that SCH patients had higher HbA1c levels than controls, but they did not conduct a detailed examination of insulin dynamics or beta-cell activity. Ogbonna et al., researching individuals with T2DM, discovered a higher prevalence of thyroid dysfunction among those with poor glycaemic control, highlighting the possibility of a bidirectional association between thyroid health and glucose metabolism [56]. These findings suggest that insulin resistance may be responsible for some of the observed HbA1c rise; however, this has not been conclusively proved due to methodological constraints in previous investigations.

**Figure 5:**
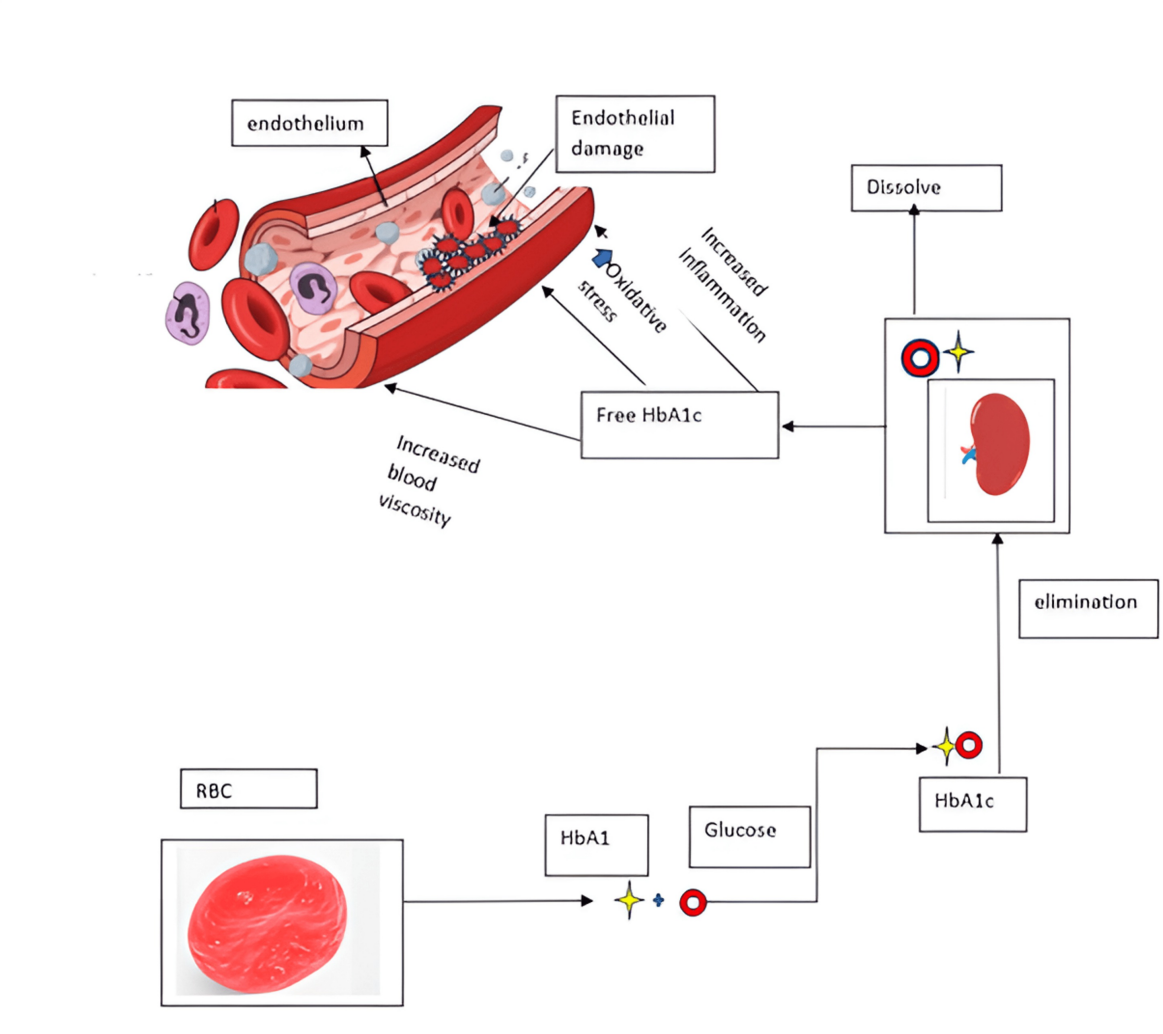
The mechanism behind the relationship between glucose metabolism, thyroid function, and cardiovascular health Haemoglobin is classified into three subgroups, the bulk of which is HbA1. HbA1c is a measure of glycaemic status in the past 2-3 months and is not affected by short-term blood glucose levels due to its constant, gradual, and irreversible reaction. HbA1c is primarily hydrolysed into plasma by the spleen. Free HbA1c can promote inflammation, oxidative stress, and blood viscosity to drive endothelial cell injury, which causes cardiovascular death. Image credit: Dr. Zareen, created in Microsoft Word (Microsoft® Corp., Redmond, WA), and pictures taken from free-to-use websites from Google (Google, Inc., Mountain View, CA).

This association is concerning, as elevated HbA1c is a known risk factor for CVD. The underlying mechanism, illustrated in Figure 5, highlights the complex relationship between glucose metabolism, thyroid function, and cardiovascular health, underscoring the importance of monitoring and managing HbA1c levels to mitigate cardiovascular risk [57].

Another important aspect is the accuracy of HbA1c as a biomarker in thyroid dysfunction. Hypothyroidism can impede erythropoiesis and extend red blood cell survival, perhaps resulting in artificially high HbA1c values that are unrelated to actual glucose levels. Both Ponnudhali and Preetha and Bhattacharjee et al.’s [54,55] research has pointed this out, suggesting that changes in erythrocyte turnover have something to do with the differences seen in SCH patients. For this, doctors should be aware when looking at HbA1c levels in this case and instead use markers such as fructosamine or glycated albumin, which are not affected by red cell turnover [58]. To minimise overtreatment or misdiagnosis, individuals with diabetes and accompanying thyroid dysfunction may benefit from continuous glucose monitoring (CGM) or alternate biochemical markers when interpreting HbA1c. Elevated HbA1c in non-diabetic persons with SCH should not be used to diagnose prediabetes without further evidence, such as FPG, OGTT, or CGM. Furthermore, regular thyroid screening in diabetes patients is recommended, especially in those who experience sudden fluctuations in glycaemic control.

The intricate relationship between TDs and cardiovascular health in diabetic patients has been a subject of extensive research. Findings suggest that thyroid dysfunction significantly increases the risk of cardiovascular complications, with a substantial number of diabetic patients remaining undiagnosed for TDs. Age and sex are pivotal factors influencing the development of TDs, with females being more susceptible. Moreover, the interplay between thyroid dysfunction and diabetes is complex, with hypothyroidism increasing cardiovascular risk and diabetes exacerbating thyroid dysfunction. Common underlying factors, including autoimmune responses and inflammation, contribute to the development of TDs in diabetic patients. Early detection and intervention are critical, emphasising the need for regular thyroid screening and monitoring of cardiovascular risk factors. Effective management of traditional risk factors and consideration of thyroid function in coronary heart disease risk assessment are also essential. Ultimately, a multifaceted approach to managing diabetes and thyroid health is vital to mitigate cardiovascular complications in affected patients.

Limitation

Notwithstanding the valuable insights gleaned from our systematic review, several limitations merit consideration. The stringent inclusion criteria, encompassing solely free full-text articles in English and human studies between a particular period only, inevitably constrained the scope of our analysis. Furthermore, the heterogeneous array of study designs, including certain study types, may be vulnerable to bias. The intricate complexity of the disease under investigation necessitated small sample sizes in numerous studies, underscoring the imperative for large-scale, rigorously designed research to yield more definitive conclusions regarding the impact of TDs on cardiac outcomes. Moreover, the pronounced heterogeneity among included studies in terms of population demographics, study design, and measured outcomes underscores the need for standardised research protocols to facilitate more accurate comparisons and generalisations. To elucidate the temporal relationship between TDs and diabetes, longitudinal studies are indispensable, and further research is warranted to decipher the underlying molecular mechanisms driving their interplay. Unravelling these mechanisms will be crucial in developing more efficacious treatment strategies and enhancing patient outcomes. By addressing these knowledge gaps, clinicians and researchers can synergise to provide more comprehensive, effective care for individuals afflicted by these intertwined conditions, ultimately optimising management and improving quality of life.

## Conclusions

Finally, this systematic review sheds light on the complex relationship between thyroid problems and DM, highlighting the crucial need for comprehensive care to avoid cardiovascular catastrophe. The interconnectedness of thyroid dysfunction and diabetes necessitates a paradigm shift in clinical management, acknowledging the critical role of thyroid function in glucose metabolism and cardiovascular health. Healthcare professionals can improve patient outcomes by taking a more holistic approach. This review's findings underscore the urgency of regular thyroid screening, cardiovascular risk monitoring, and timely intervention. As we bridge knowledge gaps and limitations, we unlock the potential for ground-breaking treatment strategies, elevating the quality of life for millions worldwide. The future of research beckons, as longitudinal studies, standardised protocols, and molecular insights promise to revolutionise the management of these intertwined conditions and pave the way for a brighter, healthier tomorrow.
